# Locally aggressive colonic and rectal cancer ,clinical trial

**Published:** 2010-08-25

**Authors:** V Radu, D Ion, MB şerban, M Ciurea

**Affiliations:** General Surgery Clinic ‘Life Memorial Hospital’, Bucharest, Emergency General Surgery 3 Clinic, Bucharest Emergency University HospitalRomania

## Abstract

**This clinical trial studies local invasions from primary colonic and rectal cancers** (urinary bladder, abdominal wall, small bowls, uterus, vagina, stomach, bile tract, spleen, duodenum, pancreas, ureters, kidneys), with or without undiscovered metastasis.

Primary locally aggressive colonic and rectal cancers include tumors that are staged T4N1–2Mx on diagnosis, and are often associated with a lower prognosis than earlier cancers. [2]

Diagnosis is based on thorough clinical evaluation, imagistic support: abdominal XR with contrast (barium enema), colonoscopy, abdominal and pelvic ultrasound exam, endoscopic endolumenal ultrasound exam, abdominal and pelvic CT / IRM with contrast (administrated both orally and intravenously), PET Scan, and intra–operatory confirmation. [3]

The primary symptom was pain.

Locally aggressive colonic and rectal cancers, primary or secondary, can extend to any visceral or parietal structure. The ability to perform a total resection is based upon anatomical localization and on the fixation of other organs to the lesion. Identifying the anatomical extension provides a better appreciation of the purpose of the tumoral resection. [1]

Radical nuanced surgery is the base of treatment of the locally aggressive colon–rectal cancer. The studies have shown that in certain localizations of the colon–rectal cancer, the locally aggressive forms can be better controlled by using multimodal therapy, including radiotherapy, either external or guided intraoperatory radiotherapy and chemotherapy with much better results. [5]

## Introduction

Colorectal cancer is the most frequent neoplasm of the gastrointestinal tract. It is the second most important cancer cause of death for both genders (after lung cancer for men and breast cancer for women) and the fourth in order of frequency after prostate cancer, breast cancer and lung cancer. [2]

Primary locally aggressive colonic and rectal cancers include tumors that are staged T4N1–2Mx on diagnosis, and are often associated with a lower prognosis than earlier cancers. [2]

Diagnosis is based on thorough clinical evaluation, imagistic support: abdominal XR with contrast (barium enema), colonoscopy, abdominal and pelvic ultrasound exam, endoscopic endolumenal ultrasound exam, abdominal and pelvic CT / IRM with contrast (administrated both orally and intravenously), PET Scan, and intra–operatory confirmation. [1]

The primary symptom was pain, associated in various degrees with digestive symptoms.

Locally aggressive colonic and rectal cancers, primary or secondary, can extend to any organ or bony structure.

## Materials and methods

The present study is a retrospective one, on a total number of 674 cases of colon–rectal cancers operated in the Emergency General Surgery 3 Clinic in the University Emergency Hospital, Bucharest, during a period of 10 years. Out of the total number of 674 cases that have been operated, 213 cases presented complicated forms, out of which only 63 showed ***locally aggressive forms*** which fulfill the inclusion criteria in our study according to the proposed theme.

The purpose of this study was to emphasize the different particularities of the local aggressive forms of the colon–rectal, linked to symptomatology, imagistic findings, intra–operatory aspects, therapeutic attitudes and post–operatory consequences.

The criteria of inclusion in the study have been the following:

1. The tumor with a colon–rectal starting point;
2. Tumoural invasion of the neighboring organs (urinary bladder, abdominal wall, small bowls, uterus, vagina, stomach, bile tract, spleen, duodenum, pancreas, ureters, kidneys);
3. Surgical treatment with radical nuance (where possible) or with a palliative nuance

The study has followed the following parameters:

1. Symptomatology;
2. Imagistic confirmation;
3. Intra–operatory aspects;
4. Therapeutic attitude

## Results and discussions

**Table 1:** Cases included

Total colonic and rectal cancers	Complicated colonic and rectal cancers	Locally aggressive forms	Other complications (perforations, haemorrhage, occlusions)
674 (100%)	213 (31,6%)	63 (9,3%)	22,1%

The cases of 674 patients with colon–rectal neoplasm interned and operated during a period of 10 years have been analyzed. Out of these 674 cases, 213 cases were complicated colon–rectal cancers; that is 31.6%, and out of these only 9.3% were locally aggressive forms (63 cases).

**Table 2:** Number of the locally aggressive colonic–rectal cancer depending on the localisation

Localisation	Nr. of cases	Localisation	Nr. of cases
Cecum and Ascending colon	12	Descending colon	2
Hepatic flexure	7	Sigmoid colon	13
Transverse colon	4	Rectal–sigmoid juncture	10
Spleen flexure	3	Rectum	12

**Figure 1:**
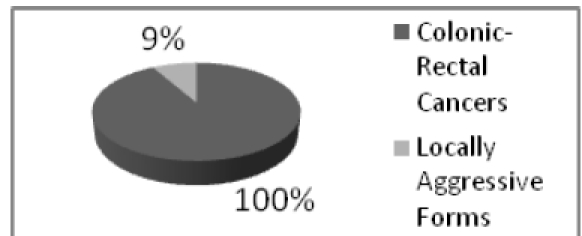
Percentage of the locally aggressive colonic–rectal cancer from all the colonic and rectal cancer studied.

Out of a total number of 674 analyzed cases only 9.3% (63 patients) corresponded to the criteria of inclusion in the study, being defined as ***locally aggressive forms of the colon–rectal cancer.***

The 63 cases of locally aggressive colon–rectal cancer are distributed as location along the entire colic frame, from the cecum to the rectum, especially at the level of the sigmoid (13 cases), the rectum (12 cases) and the cecum and the ascending colon (12 cases).

The vicinity invasion concerns almost all the abdominal viscera, with variations depending on the location of the primary tumor. Thus, in the studied lot, the most frequently invaded organ by the tumoral colon–rectal process was the urinary bladder (32%); the invasion started in the sigmoid or the rectum. The second is the abdominal wall, 25%, followed by the small bowels, 18%, uterus, 8%, and vagina, 7%. The rest of the invaded organs were stomach, bile tract, spleen, prostate, duodenum, pancreas, ureters, and kidneys. The diagnostic was clinically suggested, paraclinically upheld and confirmed by intra–operatory aspects. The clinic of the locally aggressive colon–rectal cancer was varied, including the following symptoms: modifications of the intestinal transit (diarrhea/constipation), rectorageea, vomiting, signs of neoplasic impregnation, abdominal meteorism, (32%); the invasion started in the sigmoid or the rectum. The second is the abdominal wall, 25%, followed by the small bowels, 18%, uterus, 8%, and vagina, 7%. The rest of the invaded organs were stomach, bile tract, spleen, prostate, duodenum, pancreas, ureters, and kidneys.

The diagnostic was clinically suggested, paraclinically upheld and confirmed by intra–operatory aspects.

***The clinic of the locally aggressive colon–rectal cancer*** was varied, including the following symptoms: modifications of the intestinal transit (diarrhea/constipation), rectorageea, vomiting, signs of neoplasic impregnation, abdominal meteorism, constipation, pain, pus and faecal in the urine.

**Figure 2:**
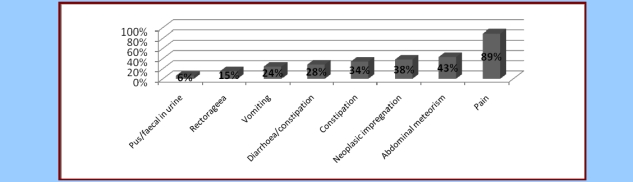
Symptoms met in locally aggressive forms of the colon–rectal cancer

The abdominal pain is the most frequent symptom in patients with colon–rectal cancer, 89% of the patients in the studied lot. The pain was diffuse in 73% of the cases, while for the rest of the cases the pain was felt as being localized at the level of the concerned colic segment.

The second place in the clinical aspect is occupied by troubles of intestinal transit (63% of the patients), manifested by constipation (34%) or by an alternation between diarrhea and constipation (28%). This element suggests the high frequency of the occlusive complications in the colon–rectal neoplasm. Diarrhea was present in two patients in the lot who also suffered from cancer of the ileocecal valve.

***The paraclinic diagnosis*** was upheld by the following investigations: abdominal echography, simple thoracic and abdominal radiography, endoscopy, irigography, computerized tomography adapted to the particularities of each case.

The most sensitive imagistic exploration is colonoscopy, which allows direct visualization of the lesion and biopsy. Only 40% of the patients in the studied lot benefitted from this investigation.

**Figure 3:**
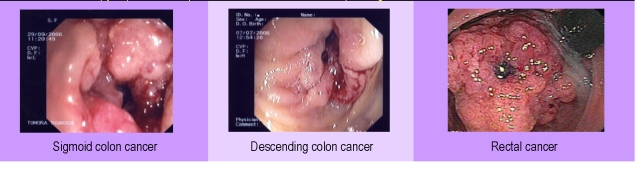
Colonoscopy aspects of complicated colonic–rectal cancer depending on the localization

Irigography can be extremely useful in diagnosing the colon–rectal cancer but it cannot differentiate the locally aggressive forms from the other forms of colon–rectal cancer. It is useful in establishing the obstruction degree of the intestinal lumen.

Thoracic radiography was performed as a routine in order to diagnose possible metastases of the lungs, while abdominal echography and computerized abdominal tomography were performed in order to estimate the size of the tumor and the possible intra–abdominal metastases.

Most of the times, the ***intra–operatory aspect ***confirms the clinic and para–clinic diagnosis, providing extra details which are necessary for the therapeutic conduct.

**Figure 4:**
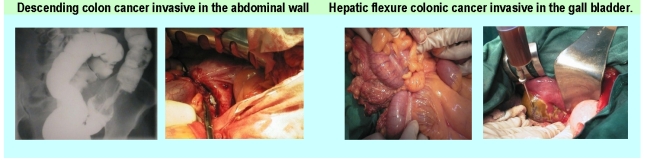
Intra–operatory aspects

The inter–visceral or visceroparietal fistulas in which one of the partners is the colon or the rectum appeared in 13 cases. These fistulas appeared as a consequence of the loco–regional extension of the tumoural process.

**Figure 5:**
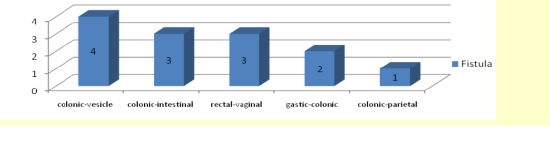
Inter–visceral or visceral–parietal fistulas

Another frequently encountered intra–operatory aspect is the perforation. In the group of the perforated clinical forms, the cases with localized peritonitis (73%) had the highest percentage. Out of the total number of cases, we have come across 5 diastatic perforations of the ascending cecum, as it follows: 2 cases at rectal–sigmoid junction, 1 case at the sigmoid colon, 1 case at the descending colon

We have come across 7 cases of synchronous cancer, as it follows: in 2 cases the localizations were at the descending colon and rectum, in 2 cases at the spleen colonic angle and sigmoid colon, in 2 cases at sigmoid colon and rectum and 1 case with synchronous cancer localized at the transverse colon. 

***The therapeutic attitude*** in the case of the locally aggressive forms of the colon–rectal cancer is influenced by the starting point of the neoplasm, its evolution stage, the general state of the patient and by the local modifications (tumor resecability, colic distension, colonic wall ischemic lesions, ileum–caecum valve integrity, etc.). [1]

The resecability is based on the anatomic location and on the adherence of other organs to the lesion. Identifying the anatomic extension offers a better estimate of the purpose of the tumoural resection.

The surgery with a radical nuance represents the base of the treatment of the locally aggressive forms of the colon–rectal cancer. However, surgery per se may lead to a higher rate of recurrences, both locally and loco–regionally. When combined with chemotherapy and radiotherapy (if such is the case), the probability of getting a resection piece with clear edges and that of the tumoural control increases significantly. [3]

Equally important is the palliative treatment in cases in which the curative treatment cannot be performed. The palliative interventions can be widely classified in non–invasive, minimally invasive and surgical. The first non–invasive palliative therapy is radiotherapy. [4]

The minimally invasive procedures usually involve mechanical techniques for reducing the symptomatology connected with pelvian tumors. These include ureteral stents for resolving the urinary stenoses and colonic metallic extensible stents or laser therapy in order to reduce the rectal obstruction. [4]

Out of a total number of 63 cases of locally aggressive forms of the colon–rectal cancer included in the study, 21 cases benefited from widened radical resections while the rest of 42 cases benefited from palliative surgical treatment.

Resection surgery was possible in 30% of the studied cases, the resecability index being inferior to the one mentioned in other studies (72%). This fact is mainly due to a late diagnosis of the illness, the patients only came to the clinic in the late, terminal stages of the illness. It should be mentioned that many of the resection operations have been performed out of necessity, being imposed by the presence of other complications that required the removing of the tumor in late oncological cases.

**Figure 6:**
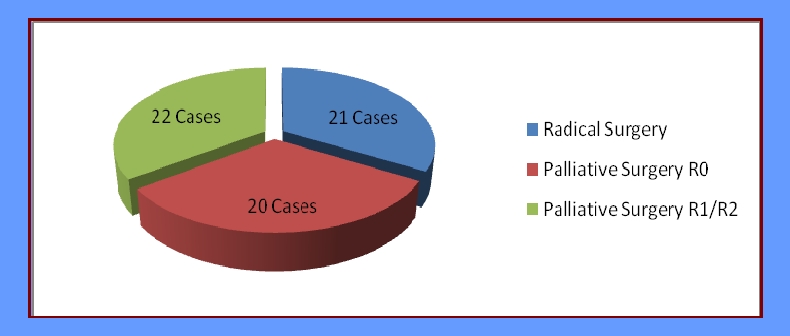
Surgical treatment of the locally aggressive colonic–rectal cancers

The surgical interventions with radical intention were possible in patients whose tumor and the tumoural invasion were localized, resecable and no dissemination existed or could be detected.

In this case, the primary tumor shows dissemination or the local aspect of the tumor or in the case the invasion did not allow a total resection, a palliative surgical intervention was taken into consideration. The R0 palliative surgical intervention implies the total removal of the primary tumor with edges of the resection piece without microscopic or macroscopic tumoural invasion. The R1 palliative surgical intervention implies the resection with tumoural edges of the visible resection piece under the microscope, while the R2 resections imply macroscopically visible edges of the resection piece.

**Table 3:** Radical surgery depending on the localization

Localisation	Cases	Localisation	Cases
Cecum and Ascending colon	5	Descending colon	1
Hepatic colonic flexure	1	Sigmoid colon	7
Transverse colon	2	Rectal–sigmoid juncture	3
Spleen colonic flexure	1	Rectum	1

The locally aggressive forms of the colon–rectal neoplasm, which benefited from radical surgery, had proximity invasions in organs such as duodenum, small bowels, bile, abdominal wall, spleen, pancreas, urinary bladder, uterus and vagina. The quantum of the invasion in the different organs was variable according to the location of the primary tumor.

Radical surgery involved the wide resection of the tumor with free resection edges, resection of the proximity invasions and that of the connected adenopathies.

In our study, there have been performed 6 right hemicolectomies, 3 abdominal wall resections, 2 duodenal excisions, 5 segmental small bawl resections, one colecistectomy, 2 segmental colectomies 2 hemigastrectomies, 2 left hemicolectomies, one splenectomy–pancreatectomy, 7 sigmoid colon resections, 3 urinary bladder resections, 5 hysterectomies, 3 rectal and sigmoid colon resections and 1 case of rectum amputation (concomitant interventions).

**Table 4:** Palliative surgery depending on the localization

Localisation	Cases	Localisation	Cases
Cecum and Ascending colon	7	Descending colon	1
Hepatic colonic flexure	6	Sigmoid colon	6
Transverse colon	2	Rectal–sigmoid juncture	7
Spleen colonic flexure	2	Rectum	11

The local aggressive forms of colorectal neoplasm that did not have surgical interventions with a radical visa were treated by palliative operations. The vicinity invasions were similar to the radical interventions.  Nevertheless, in 11 cases the invasion was multiple, including two or more organs simultaneously, according to the localization of the primary tumor.

The palliative surgical interventions had as a purpose, where it was possible, the resection as complete as possible of the primary tumor, or, where the intra–operatory aspect did not allow the resection of the primary tumor, internal derivations by ileo–transverso anastomosis, by the liberation of the intestinal transit, or external derivations, permanent colostomies, ileostomies, for the same purpose.

At the same time, internal and external urinary derivations were done in order to prevent the renal insufficiency in the cases where one of the partners involved in the tumoral process was the urinary tract.

In our study, out of the total number of 63 cases included, 30% (21 cases) could benefit from surgical treatment with a radical visa. Out of these, only 11 cases went through post –operatory systemic chemotherapy.

External radiotherapy as an aid to surgical therapy in the treatment of colorectal locally aggressive cancer has proved its utility only in low localized neoplasis, at the recto–sigmoid junction or rectum. At the same time, radiotherapy is the first and most effective form of palliative non–invasive therapy. Its benefits derive from the decrease in volume of the tumor and the improvement of obstructive syndromes, either in the digestive or in the urinary tract.

## Conclusions

Colorectal cancer is the most frequent neoplasm of the gastrointestinal tract. It is the second most important cause of cancer death for both genders (after lung cancer for men and breast cancer for women) and the fourth in order of frequency after prostate cancer, breast cancer and lung cancer.

The natural evolution of the affliction includes three phases: local invasion, lymphatic and haematic dissemination. [3]

**The local aggressivity** is the evolutionary means of a colorectal cancer, which generates complications not by the dissemination of the neoplasis, or by neoplasic impregnation manifested in a catabolic syndrome, but by the invasion of the neighboring structures (visceral, vascular, abdominal wall).

**Primary locally aggressive forms** of colorectal cancer include tumors, which are T4N1–2Mx at the time of primary diagnosis. They are often associated with a greater rate of concomitant metastasis and have, overall, poorer forecasts than early stage cancers. [2]

Despite the progress in oncologic treatment, colorectal cancer death rates have not changed significantly in recent years; surgical treatment remains the main approach. Although surgical principles are the same, a radical change of attitude in colorectal resection surgery can be seen recently, mostly as a result of the progress in anesthesia and intensive care.

In cases of advanced or recurring colorectal cancer, multimodal therapy, including radiation, surgery and chemotherapy must be used to obtain the local tumor control and to prevent or control systemic tumor dissemination, thus improving survival chances for the patient.

**Localization** is most frequent in the sigmoid (13 cases), followed by the rectum (12 cases), caecum and ascendant colon (12 cases) and recto–sigmoid junction (10 cases).

**Symptoms** of the investigated cases were varied, depending on the stage and size of the tumor, evolution phase and types of complications. The clinical diagram of the locally aggressive colorectal neoplasm includes the following symptoms: abdominal pain, abdominal meteorism, signs and symptoms related to neoplasic vicinity impregnation, modifications of intestinal transit (diarrhea/constipation), rectoragies, vomiting, pus and faecal in the urine. 

The organ most frequently invaded by the tumoural process, in the study group, was the bladder in 32% of cases, followed by the abdominal wall 25% and small intestine 18%. In 13 cases we have encountered intervisceral fistulas or visceroparietal fistulas, as a consequence of the local regional extension of the tumoural process, the most frequent being colon–urinary bladder fistula (4), colon–intestinal fistula (3) and recto–vaginal fistula (3).

Imaging investigations: Flexible colonoscopy is the most sensitive and specific means of paraclinical investigation, allowing visual exploration of the entire colon with the possibility to highlight colonic polyps, endoscopic removal of these being the means for prophylactic treatment of colon cancer. [1] Abdominal sonograms and CTs have been used to highlight the existence of tumors and potential intraabdominal metastasis.

Based on the **type of surgical treatment**, 30% of cases were dealt with by means of surgical interventions with the intent of oncologic radicality, and 70% were given palliative surgery. This has influenced the forecast, the greatest rate of survival being recorded in cases in which radical interventions were used.

**Auxiliary therapy**, as proved also by available sources, plays an important part in controlling locally aggressive forms of colorectal cancer. Post–operatory administered systemic chemotherapy has proved its effectiveness in increasing survival rates in patients operated for colon neoplasm. [5] Out of the total 63 cases included in our research, 30% (21 cases) could benefit from radical visa surgical treatment. Of these, only 11 cases followed systemic post–operatory chemotherapy. Of the other 42 cases of localized aggressive colon cancer, which received surgical palliative treatment, only 20 patients also received systemic post–operatory chemotherapy. External radiotherapy as an aid to surgical therapy in the treatment of localized colorectal aggressive cancer has proven its use only in low localized neoplasis, at the recto–sigmoid junction and rectum. At the same time, radiotherapy is the first and most effective form of palliative non–invasive therapy. Its benefits derive from the decrease in volume of the tumor and the improvement of obstructive syndromes, either in the digestive or in the urinary tract. [4]

**To conclude**, we feel that the number of cases reported in the early phases of the affliction is too small. Hence, the necessity for early diagnosis screening techniques for colorectal cancer in the early stages is important, as this will enable treatment and healing. The success in treating the ill relies on an aggressive, extensive surgical attitude, involving ‘block’ tumor removal, combined with radiotherapy and chemotherapy.
